# Eye-acupuncture with rehabilitation therapy for stroke

**DOI:** 10.1097/MD.0000000000020096

**Published:** 2020-05-01

**Authors:** Yan Shao, Pengqin Wang, Qi Wang, Lihua Yu, Lixin Zhang, Wei Wang

**Affiliations:** a Centre for Evidence-Based Chinese Medicine, Beijing University of Chinese Medicine, Beijing 100029; b Liaoning University of Traditional Chinese Medicine, Shenyang 110032; c Fuxin City Traditional Chinese Hospital, Fuxin 123000; d Shengjing Hospital Affiliated to China Medical University; e Zhongshan Hospital Affiliated to Dalian University, China.

**Keywords:** eye-acupuncture, multi-center, recovery stage, rehabilitation training therapy, stroke

## Abstract

**Background:**

Stroke survivors are accompanied by dysfunctions, greatly declining their activities of daily living and bringing burden to families and societies. Although modern rehabilitation therapy has a systematic program in post-stroke motor rehabilitation, numbers of patients still recover slowly. Eye-acupuncture (EA), as an important type of acupuncture, has been widely applied effectively in rehabilitation of stroke for about 50 years. So we combine EA with modern rehabilitation which has achieved successful results. Therefore, we need to adopt an objective and accurate evaluation method to study the effect of this method.

**Methods:**

We aim to design a multi-center, block randomized, parallel control trial to assess the effect of eye-acupuncture combined with rehabilitation training therapy for patients with hemiplegia in the convalescent stage of stroke. 360 patients will be enrolled from 6 centres, with half of them (n = 180) in the control group (routine treatment group) and others (n = 180) in the experimental group (eye-acupuncture combined with routine treatment group). Stratified block randomization will be used in the study and the serial number 001-360 which corresponds to a participant will be assigned to each center randomly. We will use the sequentially sealed envelopes to hide the generating of assignment sequence. The cases of dropouts will be recorded with reasons. And the clinical CRFs will be filled in accurately, completely, and timely for statistical analysis.

**Results:**

To verify the clinical effects, we will measure the change of bellows from visit 1 to visit 4. Primary outcomes: activity of daily living (ADL) scales (modified Barthel index); simple Fugl–Meyer motor function score; functional magnetic resonance imaging (fMRI) of the brain in the resting state. Secondary outcomes: traditional Chinese medicine (TCM) syndrome score scale; western aphasia battery (WAB); water swallow test; Montreal cognitive assessment (MoCA); growth-associated protein-43 (GAP-43); microtubule-associated protein-2 (MAP-2).

**Conclusion:**

The results of this study will provide present evidence on assessing effectiveness of EA combined with rehabilitation training for patients with hemiplegia in the convalescent stage of stroke.

**Trial registration:**

This trial has been registrated in Chinese Clinical Trail Registry with the registration number as ChiCTR1900027835 (http://www.chictr.org.cn/).

## 1 Introduction

Stroke is defined as an acute neurologic dysfunction syndrome caused by local blood circulation disorder in the brain, with high levels of morbidity, mortality, recurrence, and disability.^[1,2]^ Nowadays more patients get timely effective treatment and successfully survive after the acute stage. However, most stroke survivors are accompanied by dysfunctions in many aspects, such as dyskinesia, dysphagia, sensory, speech, and cognitive disorder, declining their activities of daily living and bringing burden to families and societies.^[3–5]^ So it is important to take comprehensive and effective rehabilitation treatment as soon as possible. Hemiplegia is one of the major post-stroke dysfunctions and has the greatest impact on activities of daily living.^[6]^ Modern rehabilitation medicine has a systematic program in post-stroke motor rehabilitation. While a large number of patients in the convalescent stage of stroke still encounter bottleneck and recover slowly or stagnantly for a long time.^[7–9]^ So we try to combine traditional Chinese medicine rehabilitation with modern rehabilitation.

Acupuncture is one of the main therapies in traditional Chinese medicine rehabilitation and clinical effectiveness has been confirmed. As a common fine-needle acupuncture therapy, eye-acupuncture is widely applied in rehabilitation of stroke.^[10–14]^ We combine eye-acupuncture (EA) with physical therapy and occupational therapy in modern rehabilitation to achieve better results and put forward an innovative method in the treatment of motor dysfunction after stroke by eye-acupuncture combined with rehabilitation training therapy. The success of rehabilitation requires effective methods and objective evaluation. The scaling method in modern rehabilitation is often applied to evaluate the effect of motor dysfunction after stroke due to its high reliability and validity. However, with the wide application of traditional Chinese medicine rehabilitation, there is still a lack of corresponding therapeutic evaluation methods and objective evaluation indicators about stroke in traditional Chinese medicine (TCM). As a result, we need to adopt an objective and accurate evaluation method to study about the effect of eye-acupuncture with rehabilitation training therapy in motor dysfunction after stroke, which may benefit the further promotion in primary hospitals.

## 2 Methods/design

### 2.1 Study design

A multi-center, block randomized, parallel control trial has been designed to assess the effect of eye-acupuncture combined with rehabilitation training therapy on limb dysfunction in patients with stroke in the convalescent stage. 360 patients will be enrolled. In order to verify and evaluate the clinical effects, outcomes will be measured by the activity of daily living (ADL) scales (modified Barthel index), modified Rankin scales (MRS), TCM syndrome score, simple Fugl–Meyer motor function score, water swallow test, western aphasia battery (WAB), Montreal cognitive assessment (MoCA) and 2 brain function remodeling indexes, growth-associated protein-43 (GAP-43), and microtubule-associated protein-2 (MAP-2) in the infarction border zone. It is expected to develop the technical standards of eye-acupuncture combined with rehabilitation training therapy, to form the characteristic TCM rehabilitation technology and diagnosis and treatment of stroke in the convalescent stage, to optimize the outcome assessment and to form the characteristic TCM rehabilitation evaluation method and hierarchical medical treatment system.

### 2.2 Technology flow chart

**Figure 1:**
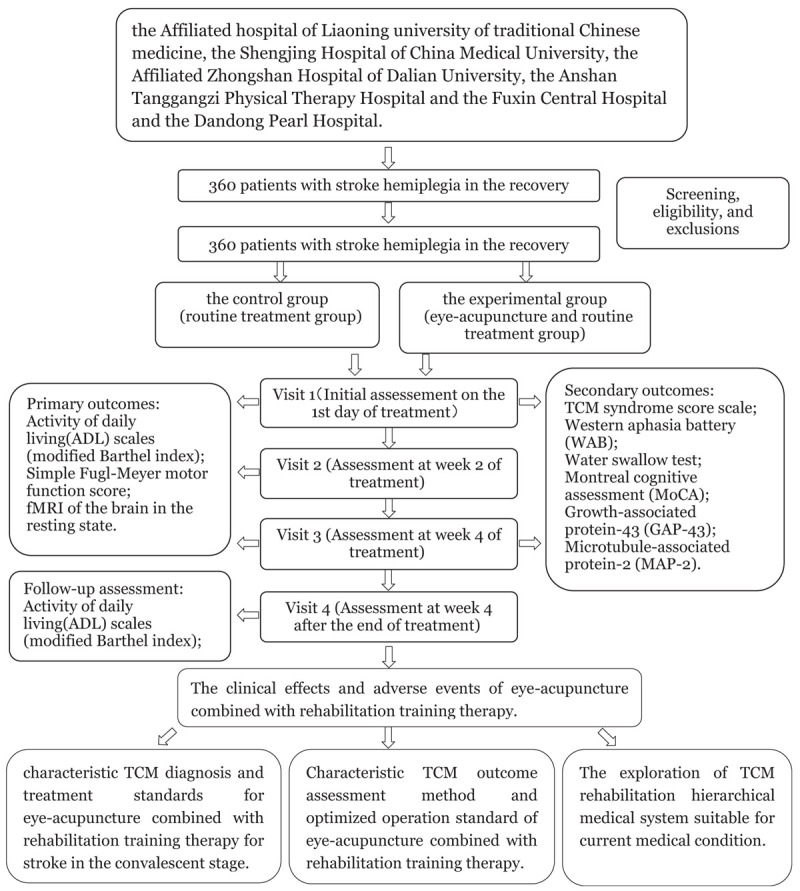
The flow chart.

The flow of the entire trial is shown in Figure 1.

### 2.3 Grouping

According to previous studies, the effective rate of the routine treatment group is 80 percent and the morbidity rate of eye-acupuncture and routine treatment group is 90 percent. Suppose *α* is 0.05 (one side) and *β* is 0.20. Calculated by PASS 11, the sample size in both groups is 157. With an estimated 10 percent dropout rate, the sample size is 175. Thus 360 patients will be recruited and divided into 2 groups in the study, with half of them (n = 180) in the control group (routine treatment group) and others (n = 180) in the experimental group (eye-acupuncture combined with routine treatment group).

### 2.4 Settings

The lead center of the study is the Affiliated Hospital of Liaoning University of Traditional Chinese Medicine and the branch centers include the Shengjing Hospital of China Medical University, the Affiliated Zhongshan Hospital of Dalian University, the Fuxin Central Hospital, the Anshan Tanggangzi Physical Therapy Hospital, and the Dandong Pearl Hospital (described in Table 1).

**Table 1:**
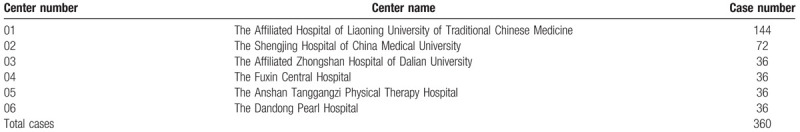
The specific case number in 6 centers.

### 2.5 Randomization

Stratified block randomization will be used in the study based on the SAS proc plan sentence at the appropriate length. The seed is set as the running time of the program and the case number of the experimental group and the control group is in a ratio of 1:1. The serial number 001-360 which corresponds to a participant will be assigned to each center randomly. Centers are ordered in the alphabetical list by the initial letter of pinyin and offer treatment in increasing sequence.

### 2.6 Concealment

Assignment sequence generated by random hiding is put into the sequentially encoded, sealed, and opaque envelope. Researchers will open the envelopes in sequence and assign participants into different groups.

### 2.7 Cases of dropouts

1. When participants drop out of the study, researchers should try to contact them, ask for reasons, record the last time of medication, and finish the evaluation items.
2. When participants drop out because of adverse events, researchers should take appropriate treatment and closely follow them up until the adverse events disappear or recover. Adverse events should be recorded and filled out in a case report form (CRF) and carried out statistical analysis.
3. Researchers should record the main reasons for dropout.
4. Whether participants drop out or not, all that have been recruited and have used the treatment number should be kept as records, which can be analyzed by intention-to-treat (ITT) analysis. There is no need to add cases after dropouts and all CRFs should be submitted to the statistical unit to make the analysis. The dropout rate of this clinical study should be under 10 percent.

### 2.8 Data management

1. Clinical CRFs should be filled in accurately, completely, and timely by clinical researchers and the original records should be kept.
2. To ensure the facticity and validity of the data, participants are asked to note the date and the total time they fill in the forms. The basic questionnaire survey should be completed within 14 days before the treatment starts and the information after enrollment should be completed within ±2 days of the stipulated date.
3. To reduce dropouts and improve compliance, researchers and data collectors should remind the participants 1 day before the stipulated visit date.
4. Data entry and modification: data entry and management are in the charge of data administrators from the statistical unit. Two data administrators input independently and proofread to make sure the accuracy of the data.
5. Question management: data administrators make DRQ from the questions existed in the CRFs and send them to researchers. Researchers should answer and return DRQs as soon as possible. Then data administrators will modify, confirm and enter the data again according to the answer. The DRQs can be sent again if it is necessary.
6. Data auditing and caging: clinical researchers, data administrators, and statistical analysts should audit the established database by the end of the study. After blind review and confirmation, the main researchers and statistical analysts will cage the data and the caging data file should not be modified.

### 2.9 Statistical analysis

1. Full analysis set (FAS): FAS refers to the set of participants as close as possible to the intention-to-treat (ITT) analysis, including qualified cases and dropout cases.
2. Per-protocol set (PPS): PPS, also known as effective cases or samples, is the set of participants who fully comply with the research protocol and complete stipulated information collection. PPS is a subset of FAS.
3. Safety set (SS): SS refers to the set of participants who have received treatment at least one time. SS is used for security analysis.

## 3 Objects/participants

Given that stroke in the convalescent stage is characterized by long course and relatively stable condition, a multi-center, randomized controlled trial has been designed to evaluate the clinical effect of eye-acupuncture combined with rehabilitation training therapy.

### 3.1 Enrollment and recruitment of participants

1. Enrollment: subjects of the study will be recruited through television, radios, newspapers, communities, and hospitals on the principle of respecting subjects’ privacy and voluntary participation.
2. Inclusion criteria: researchers in each subcenter are responsible to introduce the objectives, design, process, benefits, risks, and compensation in this study to subjects. According to the diagnostic criteria, inclusion criteria and exclusion criteria, researchers conduct preliminary screening for the recruited subjects. Those who meet the criteria are subjects who may participate in the study. They will sign the informed consent and their name, gender, age, address, telephone number, and other information should be recorded in detail.
3. Screening: subjects who may participate in the study are further screened according to the diagnostic criteria, inclusion criteria, and exclusion criteria. Those who have met the criteria and signed the informed consent will conduct the basic questionnaire survey within 14 days before the treatment period. Subjects who can complete the questionnaire under guidance (more than 80% completion without logical problem) will be screened by physical and chemical examination indicators. The qualified ones will participate in clinical trials.

### 3.2 Diagnostic criteria

#### 3.2.1 Traditional Chinese medicine diagnostic criteria

Taking diagnosis and standard for evaluating curative effect formulated by the state administration of traditional Chinese medicine acute encephalopathy team as reference.

##### 3.2.1.1 Diagnostic criteria for stroke

Primary symptom: hemiplegia, unconsciousness, sluggish speech, hemianesthesia, and deviation of the eye and mouth.
Secondary symptoms: headache, vertigo, changes in pupil and spirit, choking when drinking water, strabismus, and ataxia.
Acute onset with inducement and often premonitory signs.
Most of the patients are over 40 years old.
Patients matching more than 2 primary symptoms or 1 primary symptom along with 2 secondary symptoms can be diagnosed in combination with the onset, inducement, premonitory signs, and age; the diagnosis can also be made with imaging examination.

##### 3.2.1.2 Staging criterion

The acute stage: within 2 weeks after stroke attacking viscera, no more than 1 month after stroke attacking meridians.
The convalescent stage: 2 weeks to 6 months after the onset.
The sequela stage: 6 months after onset.

#### 3.2.2 Western medicine diagnostic criteria

##### 3.2.2.1 Diagnostic criteria for cerebral infarction

Taking guidelines for diagnosis and treatment of acute ischemic stroke in China in 2010 as reference.

3.2.2.1.1 Diagnostic criteria

Acute onset;
The focal neurological deficit, limited numbers of comprehensive neurological deficit;
Signs and symptoms lasting more than 24 hours;
Exclusion of non-vascular encephalopathy;
Images of brain CT or MRI show ischemic duty focus and exclusion of cerebral hemorrhage and other diseases.

3.2.2.1.2 Classificatory diagnosis

There are kinds of clinical pathology types due to the difference in the location and size of cerebral infarction, collateral compensative capacity, secondary cerebral edema, and so on. As their treatment is very different, rapid and accurate classificatory is required in the acute stage, especially in the ultra-early stage (within 3–6 hours).

3.2.2.1.2.1 Clinical classification

Oxfordshire community stroke project (OCSP) classification: patients can be classified rapidly according to clinical manifestations instead of imaging results, which is ahead of conventional CT and MRI and indicates the size and location of occluded vessels and infarcted lesions. This method is easy for the clinical application and has important significance in guiding treatment and evaluating prognosis.

OCSP clinical classification criteria

Total anterior circulation infarction (TACI): it is shown as triad syndrome, that is, complete middle cerebral artery (MCA) syndrome, including dysfunction of higher neurological activity in the brain (consciousness disorder, aphasia, miscalculation, spatial orientation disorder, and so on), homonymous hemianopia, the more serious motor and/or sensory disorders in the opposite 3 parts (face, upper limb, and lower limb). Most of the cerebral infarction is caused by the occlusion of proximal MCA and a few are internal carotid artery siphon.

Partial anterior circulation infarcts (PACI): it is shown as 2 of the above triad syndrome, or only dysfunction of high-level neurological activity in the brain, or sensorimotor deficit limited than TACI, which indicates that it is caused by the middle or minor occlusion of distal MCA and branches or ACA and branches.

Posterior circulation infarcts (POCI): it is shown as various degrees of the vertebrobasilar syndrome, including ipsilateral cerebral nerve paralysis and contralateral sensorimotor dysfunction, bilateral sensorimotor dysfunction, binocular coordination, and cerebellar dysfunction without long bundle syndrome or visual field defect. Different size of the brainstem and cerebellar infarction is caused by the occlusion of vertebral–basilar arterial and branches.

Lacunar infarction (LACI): it is shown as the lacunar syndrome, such as pure motor hemiplegia, pure sensory stroke, ataxia hemiplegia, and dysarthric-clumsy hand syndrome. Most of the small lacunar infarcts are caused by lesions of basal ganglia or small perforating branches of pontine.

Posterior circulation infarction (POCI): various vertebrobasilar syndrome: ipsilateral cerebral palsy and contralateral sensorimotor dysfunction; bilateral sensorimotor dysfunction; binocular cooperative activity and cerebellar dysfunction; no long bundle syndrome or visual field defect. It is caused by occlusion of the vertebrobasilar artery and branches. Lacunar infarction (lacI): lacunar syndrome, such as pure motor hemiplegia, pure sensory stroke, ataxia hemiplegia, hand clumsiness dysarthria syndrome, etc. Most of them are small lacunar lesions caused by the small perforating branches of basal ganglia or pons.

3.2.2.1.2.2 Structural imaging (CT) classification

Large infarction: more than 1 lobe with a maximum cross-sectional diameter of more than 5 cm.
Middle infarct: less than 1 lobe with a maximum cross-sectional diameter of 3.1 to 5 cm.
Small infarction: maximum cross-sectional diameter of 1.6 to 3 cm.
Lacunar infarction: maximum cross-sectional diameter of less than 1.5 cm.

##### 3.2.2.2 Diagnostic criteria for intracerebral hemorrhage (ICH)

Taking the diagnostic criteria of ICH in guidelines for diagnosis and treatment of ICH as reference.

Combined with CT and other imaging examinations, ICH is easy to be diagnosed based on the clinical signs and symptoms, such as sudden onset, severe headache, vomiting, and neurological dysfunction. However, there is no gold standard for the diagnosis of primary cerebral hemorrhage, especially hypertensive cerebral hemorrhage. So it is necessary to exclude all kinds of secondary cerebral hemorrhage to avoid misdiagnosis. Making the final diagnosis need to meet all the following criteria:

A definite history of hypertension;
Typical bleeding sites (including basal ganglia, ventricles, thalamus, brainstem, and cerebellar hemisphere);
Excluding secondary cerebral vessels disease with DSA/CTA/MRA;
Excluding diseases like the brain tumor or cavernous malformations (CM) with enhanced MRI examination in the early stage (within 72 hours) or the late stage (3 weeks after the disappearance of hematoma);
Excluding various coagulopathy.

### 3.3 Inclusion criteria

Age 30 to 75 years;
Meet the diagnostic criteria for cerebral infarction and intracerebral hemorrhage;
Meet the diagnostic criteria for stroke and staging criterion in traditional Chinese medicine,
The onset time of cerebral infarction or intracerebral hemorrhage is within 3 months; the evaluation of modified Barthel index is less than 70 points, and the evaluation of modified Rankin scale is more than 3 points;
Modified Barthel index < 70, MRS ≥ 3;
Patients and his family members both voluntarily agree to participate in this clinical trial and sign the informed consent.

### 3.4 Exclusion criteria

(1) Lacking definitive imaging examination, such as CT or MRI.
(2) Special types of stroke: individuals with progressive stroke or intracerebral hemorrhage after cerebral infarction or with disturbance of consciousness.
(3) Individuals with mild syndrome; individuals with slight stroke or slight neurological defect and the evaluation of modified Barthel index is more than 70 points; individuals without disability or with rapid improvement of symptoms, such as individuals with the transient ischemic attack (TIA) and (RIND).
(4) Individuals who receive treatment of dredging blocking vessels in the acute stage (such as thrombolysis, arterial thrombectomy, super early thrombus aspiration, and stenting).
(5) Individuals with bleeding tendency; individuals with serious bleeding within 3 months.
(6) Individuals with severe cardiac insufficiency, atrial fibrillation, heart valve disease, or individuals after heart valve replacement.
(7) Individuals with other diseases, which can influence the function of limb movement; individuals with limb motor dysfunction caused by claudication, rheumatoid arthritis, or gouty arthritis before treatment.
(8) Similar diseases: individuals with CT or MRI imaging of neurological symptoms caused by non-vascular factors; individuals who have suffered from intracranial neurobiology or arteriovenous malformation, aneurysm or neuro-tumor, or individuals with cranial neuro-tumor visible in imaging; individuals with cerebral trauma, multiple sclerosis, cerebral parasites, seizures, hysterical paralysis, or cerebral amyloid angiopathy.
(9) Individuals may be exposed to risks or damage by participating in this study from recruitment investigators’ points of view.
(10) Disabled individuals (blind, deaf, dumb, mental disorders, mental disorders, and any other limb disability which can affect the evaluation of neurological impairment).
(11) Individuals who have known or suspected of having a history of drug or alcohol abuse, or considered inappropriate by the recruitment investigators.
(12) Allergic individuals: people who have known or suspected of being allergic to related substances in this study.
(13) Individuals who are in other clinical trials or have participated in other drug trials in the past 3 months.

### 3.5 Informed consent (ethical considerations)

This trial is designed in accordance with the principles of the Declaration of Helsinkiand and relevant Chinese clinical trial research criteria and regulations. The trial cannot be implemented until the research project and the ethics committee of each research units consider, agree, and sign the approval opinions. During the clinical trial, any modification of the protocol should be approved by the ethics committee. Prior to enrollment, clinical research physicians should fully introduce the objective, process, and possible risks of the study to each patient or his designated agent, along with a written informed consent. The trial cannot be carried out until the patient or his agents or family members signs the informed consent.

### 3.6 Collection and preservation of the baseline data

We have established the clinical research regulation, standardized operating procedure, data collection, and analysis of the multi-center randomized blind controlled trial. We will collect and evaluate 360 cases of patients with cerebral infarction or intercerebral hemorrhage no more than 3 months and the treatment will last 4 weeks.

## 4 Intervention

Participants are divided into 2 groups, the control group (routine treatment group) and the experimental group (eye-acupuncture and routine treatment group).

### 4.1 Normalized operation of eye-acupuncture combined with rehabilitation training therapy

**Figure 2:**
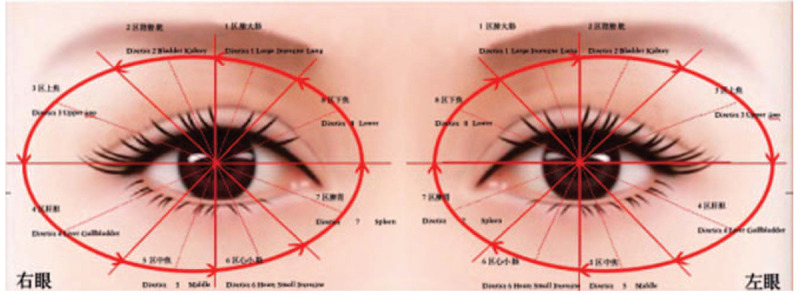
Diagram of acupoint location of eye-acupuncture.

The rehabilitation programs are determined according to the patient's condition and the evaluation of rehabilitation. Based on the principle for the selection of acupoints, patients receive eye-acupuncture treatment and keep the needles retained in the process of rehabilitation. The rehabilitation training is carried out by physical therapists and the program includes eye-acupuncture physical therapy, eye-acupuncture occupational therapy, eye-acupuncture speech training, eye-acupuncture cognitive rehabilitation training, eye-acupuncture cognitive rehabilitation training, and eye-acupuncture pain rehabilitation techniques. The needles are withdrawn in 5 minutes after the rehabilitation. Researchers should rotate the needle with the thumb and forefinger lightly and slowly until half of the needle is pulled out. Then withdraw the needle slowly and press the hole with a dry cotton ball after pulling out the needle immediately. Pick up the needle and hold the handle with the thumb and food fingers of the needle-holding hand. After gently turning, slowly pull out the needle 1/2, and then slowly pull out the needle (shown in Fig. 2).

1. *Eye-acupuncture physical therapy*: Eye-acupuncture physical therapy includes eye-acupuncture exercise therapy and eye-acupuncture equipment training. Needles are embedded in the liver region, kidney region, upper-jiao region, and lower-jiao region on both sides. The rehabilitation programs are determined by functional evaluation. Participants are expected to recover or improve dysfunction by taking various kinds of exercise training with hands or equipment, which include limb intelligent feedback training system, gait analysis treadmill, intelligent sports training device, electric erecting bed training, dysphagia therapeutic apparatus, scene interactive rehabilitation system, balance function examination training system, and so on.
2. *Eye-acupuncture occupational therapy*: Needles are embedded in the liver region, kidney region, upper-jiao region, and lower-jiao region on both sides of the eyes. At the same time, participants get training and treatment by choosing purposeful activities based on their needs in daily life, family life, social intercourse, and career.
3. *Eye-acupuncture speech training*: Needles are embedded in the upper-jiao region, lower-jiao region, heart region, and spleen region on both sides of the eyes for participants with speech disorder. At the same time, conducting the conventional speech training
4. *Eye-acupuncture cognitive rehabilitation training*: Needles are embedded in the upper-jiao region, lower-jiao region, heart region, and kidney region on both sides of the eyes. At the same time, rehabilitation treatment for attention, memory, calculation, thinking, the ability to solve problems and execute, and perceptual disorder is implemented in order to improve cognitive function and activity of daily living.
5. *Eye-acupuncture pain rehabilitation techniques*: Needles are embedded in the upper-jiao region, lower-jiao region, heart region, and spleen region on both sides of the eyes. At the same time, participants get rehabilitation training based on their major dysfunction.
6. *Needles selection*: Specialized needles for eye-acupuncture combined with rehabilitation training therapy at the length of 0.30 mm × 8 mm, 0.25 mm × 8 mm, 0.30 mm × 7 mm, 0.25 mm × 7 mm (Patent Number: CN201320166807).
7. *Position selection*: in the supine position.

### 4.2 Normalized operation and acupoint selection in body acupuncture

Upper limbs: Jianyu (LI15), Quchi (LI11), Shousanli (LI10), Waiguan (TE5), Hegu (LI4), Yangchi (TE4), Zhongzhu (TE3).

Lower limbs: Huantiao (GB30), Weizhong (BL40), Weiyang (BL39), Yanglingquan (GB34), Zusanli (ST36), Sanyinjiao (SP6), Jiexi (ST41), Kunlun (BL60).

(Taking the recovery stage of stroke in the fifth edition of acupuncture and moxibustion, a textbook for TCM planning in colleges and universities, as reference.)

### 4.3 Therapeutic schedule

Participants are randomly divided into 2 groups and receive different treatments after enrollment.

Basic treatment in 2 groups: taking standardized treatment and the relevant contents in Chinese guidelines for the prevention and treatment of cerebrovascular diseases 2010 and Chinese guidelines for the diagnosis and treatment of cerebral hemorrhage 2014.

The control group: carrying out routine rehabilitation training (for example, physical therapy, occupational therapy, cognitive training, speech training, and swallowing training) on the basis of basic treatment. If participants need body acupuncture treatment, referring to the normalized operation and acupoint selection in body acupuncture above-mentioned.

The experimental group: carrying out eye-acupuncture combined with rehabilitation training (for example, physical therapy, occupational therapy, cognitive training, speech training, and swallowing training). The time of training with needles in the eyes should be less than 6 hours. Needles need to be withdrawn after training. If participants need body acupuncture treatment, referring to the normalized operation and acupoint selection in body acupuncture above-mentioned.

### 4.4 The course of the treatment

All participants will receive treatment once a day for 5 consecutive days and rest for 2 days a week. The treatment will last for 4 weeks in total.

## 5 Effect measurement

### 5.1 Therapeutic outcomes

#### 5.1.1 Primary therapeutic outcomes

ADL scales (modified Barthel index);
Simple Fugl–Meyer motor function score;
Functional magnetic resonance imaging (fMRI) of the brain in the resting state.

#### 5.1.2 Secondary therapeutic outcomes

TCM syndrome score scale;
WAB;
Water swallow test;
MoCA;
GAP-43;
MAP-2.

### 5.2 Therapeutic mechanism outcomes

#### 5.2.1 Criterion of primary therapeutic outcomes

1. The comparison of obvious effective rate and effective rate in improvement degree of motor function and living ability among groups and within groups.
2. The comparison of before-and-after treatment in fMRI of the brain in the resting state.

*Applying Nimodipine methods*:
Clinical control rate: n ≥ 90%
Obvious effective rate: 90% > n ≥ 70%
Effective rate: 70% > n ≥ 30%
Ineffective rate: n < 30%
n = [(points before treatment − points after treatment) ÷ points before treatment] × 100%, n is expressed as a percentage.

#### 5.2.2 Criterion of secondary therapeutic evaluation index/criterion of secondary therapeutic outcomes

1. Activity of daily living (ADL) scales (modified Barthel index); WAB; water swallow test; MoCA; GAP-43; MAP-2.
2. The quantified and classified standard for TCM symptoms
  *Effectivity*: the symptoms and signs in TCM improve and the score n ≥ 50%;
  *Inefficiency*: the symptoms and signs in TCM do not obviously improve, or even become worse, and the score n < 50%.
  n = [(total score before treatment − total score after treatment) ÷ total score before treatment] × 100%.
3. The comparison of obvious effective rate and effective rate among groups and within groups made by ADL scales (modified Barthel index), WAB and water swallow test; the comparison of before-and-after treatment in GAP-43 and MAP-2.

### 5.3 Discontinuation criteria

Discontinuation means that clinical trials have not been completed as planned and part of them have been stopped halfway. The purpose is to ensure the interests of the subjects, protect the quality of the trials, and avoid unnecessary economic losses.

The trial should be discontinued timely if serious safety problems happened.

The trial should be discontinued if the treatment is poor or ineffective, or with no clinical value in order to avoid delaying the effective treatment of the subjects and unnecessary economic loss;

The subject is not willing to continue the clinical trial and drops out halfway;

The subject with allergic reactions or other adverse reaction is supposed to drop out of the trial if researchers think it necessary.

Researchers should keep a record of the reasons subjects drop out in detail.

Automatic withdrawal (subjects who cannot insist on the treatment);
Adverse events;
Serious complication;
Symptoms deteriorate and emergency measures must be taken.

### 5.4 Measurement, records, time, and repetitions

The curative effect is assessed at baseline, 0, 2, and 4 weeks after each treatment and 4 weeks after the end of treatment during the follow-up period. Investigators will fill in the complete CRF forms and fax them to the clinical trial center and data management center in time.

Primary outcome measures: the first day of diagnosis, the 14th and the 28th day after treatment.

Secondary outcome measures: the first day of diagnosis, the 14th and the 28th day after treatment. The subjects are followed up for 4 weeks after treatment.

#### 5.4.1 Visit 1 (Day-3∼0)

The basic demographic information, past medical and treatment history, course of the disease, complication, medication, and so on.
General vital signs: temperature, respiration, heart rate, and blood pressure (measured after sitting still for 5 minutes).
General physical examination and specialist examination.
Laboratory examination: blood routine examination (red blood cell count, white blood cell count, platelet count, hemoglobin), urine routine examination, 4 coagulation tests (prothrombin time, activated partial thromboplastin time, plasma fibrinogen, and thrombin time), and 12-lead ECG examination.
fMRI of the brain in the resting state and GAP-43 and MAP-2.
Primary and secondary outcomes scale evaluation.
Qualified patients (meeting all inclusion criteria and failing to meet all exclusion criteria) are randomly divided into groups based on the random number and they are informed of the treatment method.
After the first treatment, the experimental group has an fMRI of the brain in the resting state.

#### 5.4.2 Visit 2 (14th day after the treatment)

Keep a record of general vital signs: temperature, respiration, heart rate, and blood pressure (measured after sitting still for 5 minutes).
General physical examination and specialist examination.
Primary and secondary outcomes scale evaluation.
Keep a record of adverse events and drug use.

#### 5.4.3 Visit 4 (28th day after the treatment)

Keep a record of general vital signs: temperature, respiration, heart rate, and blood pressure (measured after sitting still for 5 minutes).
General physical examination and specialist examination.
Laboratory examination: blood routine examination (red blood cell count, white blood cell count, platelet count, hemoglobin), urine routine examination, 4 coagulation tests (prothrombin time, activated partial thromboplastin time, plasma fibrinogen, and thrombin time), and 12-lead ECG examination.
fMRI of the brain in the resting state and GAP-43 and MAP-2.
Primary and secondary outcomes scale evaluation.
Keep a record of adverse events and drug use.

### 5.5 Blinding test

No.

### 5.6 Follow up

4-week follow-up by telephone after treatment. ADL scales (modified Barthel index).

## 6 Safety and adverse events

### 6.1 Indexes, time, and methods of safety

Blood routine examination (red blood cell count, white blood cell count, platelet count, hemoglobin), urine routine examination, 4 coagulation tests (prothrombin time, activated partial thromboplastin time, plasma fibrinogen, and thrombin time), and 12-lead ECG examination.

### 6.2 Report form

All information about adverse events, whether mentioned by subjects, found by investigators, or assessed by physical examination, laboratory examination or other methods, should be recorded on the case report form.

### 6.3 Breaking blinding and treatment in emergency

When serious adverse events happen, researchers should break blindness and take symptomatic treatment immediately.

### 6.4 The definition of serious and non-serious adverse events

#### 6.4.1 Adverse events

Adverse events cover all the occurrence of deterioration of symptoms and syndrome. It also includes relevant clinical conditions found in the laboratory or any process of diagnoses, such as unscheduled treatment measures, withdrawal from the trial, or the abnormal laboratory examination items exceeding the norm for 20%. Adverse events may include new disease, deterioration of symptoms or signs during treatment, the effect of drugs, unrelated to participation in the trial, and a combination of 1 or more factors. Therefore, the term adverse event does not mean causality with the intervention.

#### 6.4.2 Serious adverse events

Adverse events occur at any dose of the test drug or at any time during the observation period, including death, immediate danger to life, the requirement to be in hospital or prolong hospitalization, disability, and congenital malformation; important medical significance (it refers to those events that will not immediately endanger life or cause death or need hospitalization, but may do harm to patients and need measures to prevent) and medical treatment needed to avoid permanent injury or damage.

#### 6.4.3 Severity levels of adverse reactions

Mild: it is usually transient and does not affect daily activities.
Moderate: it is quite uncomfortable and affects daily activities.
Severe: it makes patients unable to carry out daily activities.

### 6.5 Observation and records of adverse reactions

The adverse events record form is set in the CRFs, where researchers are required to fill in the time, severity, duration, measures, and outcomes of adverse events truthfully and to estimate the relationship between adverse events and trials based on 5-level judgment standard.

#### 6.5.1 Evaluation criteria for adverse events (including symptoms, signs, and test indexes)

The occurrence time of adverse events coincides with the treatment time;
The adverse events are related to the known adverse reactions of the treatment;
The adverse events cannot be explained by other reasons;
The adverse events will be relieved or disappear after treatment ends;
The adverse events will reappear after treating it again.

#### 6.5.2 Criteria for adverse reactions

1. *Definitely related*: The occurrence of the reaction conforms to the reasonable time sequence after medication and the known reaction type of the suspected treatment method. The reaction is improved after discontinuation but reappears after treatment again.
2. *Probably related*: The occurrence of the reaction conforms to the reasonable time sequence after medication and the known reaction type of the suspected treatment method. The patient's clinical state or other treatment methods may also lead to the same reaction.
3. *Probably not related*: The occurrence of the reaction is not quite consistent with the reasonable time sequence after medication and the known reaction type of the suspected treatment method. The patient's clinical state or other treatment methods may also lead to the same reaction.
4. *Definitely not related*: The occurrence of the reaction does not meet the reasonable time sequence after medication and some of the reactions cannot be explained by the treatment in the trials. The reaction may take place because of the patient's clinical state or other treatment methods, disappear when the state improves or treatment ends and reappear when using other methods repeatedly.
5. *Unknown*: The occurrence of the reaction does not have a definite relation with the time sequence after medication. Both the suspected and other treatments in the trial can lead to the reaction.

The definitely related, the probably related and the unknown can be regarded as adverse reactions of the treatment.

During the trial, researchers are required to observe and fill in the time, severity, duration, measures, and outcomes of any adverse events carefully and to follow up properly. If an abnormal value with clinical significance is found, it should be observed at least once a week until it reaches the normal or baseline level.

### 6.6 Report on adverse events

If serious adverse events happen in the trial, the unit undertaking the clinical study should take necessary measures immediately to keep the safety of the subjects and report to the local provincial drug administration and the State Food and Drug Administration (Tel of drug research supervision office of safety supervision department: 01068313344-10131023), the sponsor and the ethics committee in the unit in charge of the study (Tel: 024-31961989), and notify all participating units within 24 hours. The sponsor will ensure to meet all reporting procedures required by laws and regulations.

### 6.7 Treatment methods of adverse events

All the information on serious adverse events should be collected and recorded in the serious adverse events record form. Necessary measures should be taken immediately to ensure the safety of the subjects when each case of adverse events happens, which should be reported to the ethics committee of the Affiliated Hospital of Liaoning University of Traditional Chinese Medicine.

Common adverse reactions in the process of acupuncture include fainting, bent needles, stuck needles, broken needles, and hematoma. When the patient faint during the acupuncture, stop needling immediately, withdraw all the needles, keep the patient lie flat, and pay attention to keep him warm. In mild conditions, patients can recover after lying on his back for a while without the pillow and drinking warm water or with sugar. In serious conditions, besides the above treatment, patients will get acupuncture at Renzhong (GV26), Suliao (DU25), Neiguan (PC6), and Zusanli (TE3) and get moxibustion at Baihui (GV20), Guanyuan (CV4), and Qihai (CV6). If the patient is still unconscious with weak breath and pulse, emergency measures should be taken into consideration.

When the needle is bent, do not withdraw the needle forcibly in case of breaking the needle and leaving it in the body. If the handle is slightly bent, the needle should be withdrawn slowly; if the angle of bending is too large, the needle should be withdrawn along the bending direction. If the bending is caused by the change of the position, the patient should be slowly back to the original position and the needle should be slowly withdrawn after the local muscles are relaxed.

If the needle is stuck, percuss the needle handle or press the muscles near the acupoints of the needle, or insert another needle nearby. If the sticking of the needle is caused by excessive rotation in 1 direction, twist it back in the opposite direction. Scraping or percussing the needle handle also helps to release the twisted muscle fiber.

The patient cannot change his original position when the needle is broken. If part of the needle body is exposed outside the body, it can be pulled out with fingers or forceps. If the broken end is flat with the skin or slightly sunk, press the skin around the needle vertically with the thumb and the index finger of the left hand until the broken end is exposed, then remove it with forceps held in the right hand. If the broken needle completely penetrates into the subcutaneous or deep muscle layer, it should be located with an X-ray and removed by surgery.

A small part of subcutaneous hemorrhage or local bruises can heal themselves without treatment. If the local swelling and pain are severe, or the bruises affect the daily activity function, stop bleeding with cold compress first and then do hot compress or gently massage in the local area to promote the local blood stasis dissipation and absorption.

## 7 Key technology

Eye-acupuncture combined with rehabilitation training therapy is aimed at the dysfunction in patients in the convalescent stage of stroke. In order to improve the limb motor function and activity of daily living, different eye-acupuncture regions are selected based on corresponding TCM syndrome differentiation and the stage of stroke, which is the key to the technology. Eye-acupuncture requires skillful manipulation and strict disinfection. When combining eye-acupuncture with physical therapy and occupational therapy, needles are not allowed to fall out in case of bleeding in eye-acupuncture regions around the eyes. The key to ensuring the curative effect of eye-acupuncture combined with rehabilitation training therapy is to establish the normalized operation of eye-acupuncture and rehabilitation training.

## Author contributions

**Conceptualization:** Yan Shao, Pengqin Wang.

**Data curation:** Yan Shao, Lihua Yu, Lixin Zhang, Wei Wang.

**Formal analysis:** Yan Shao, Wei Wang.

**Funding acquisition:** Yan Shao, Pengqin Wang, Lihua Yu, Lixin Zhang, Wei Wang.

**Investigation:** Qi Wang, Wei Wang.

**Methodology:** Qi Wang.

**Project administration:** Yan Shao, Pengqin Wang, Lihua Yu, Lixin Zhang, Wei Wang.

**Resources:** Qi Wang, Lihua Yu, Lixin Zhang.

**Software:** Qi Wang.

**Supervision:** Yan Shao, Pengqin Wang, Lihua Yu, Lixin Zhang, Wei Wang.

**Validation:** Yan Shao, Lixin Zhang.

**Visualization:** Qi Wang.

**Writing – original draft:** Qi Wang.

**Writing – review & editing:** Qi Wang.
